# Antifungal activity of caspofungin in experimental infective endocarditis caused by *Candida albicans*

**DOI:** 10.1590/0074-02760160494

**Published:** 2017-03-27

**Authors:** Gerardo Becerra Victorio, Lorena Michele Brennan Bourdon, Leonel García Benavides, Selene G Huerta-Olvera, Arturo Plascencia, José Villanueva, Erika Martinez-Lopez, Iván Isidro Hernández-Cañaveral

**Affiliations:** 1 Universidad Autónoma de ChiapasUniversidad Autónoma de ChiapasFacultad de Ciencias QuímicasChiapasMexicoUniversidad Autónoma de Chiapas, Facultad de Ciencias Químicas, Chiapas, Mexico; 2 Comisión para la Protección contra Riesgos Sanitarios del Estado de JaliscoGuadalajaraMexicoComisión para la Protección contra Riesgos Sanitarios del Estado de Jalisco, Guadalajara, Mexico; 3 Universidad de GuadalajaraUniversidad de GuadalajaraCentro Universitario de Ciencias de la SaludDepartamento de FisiologíaGuadalajaraJaliscoMexicoUniversidad de Guadalajara, Centro Universitario de Ciencias de la Salud, Departamento de Fisiología, Guadalajara, Jalisco, Mexico; 4 Universidad de GuadalajaraUniversidad de GuadalajaraCentro Universitario de la CiénegaDepartamento de Ciencias Médicas y de la VidaOcotlanJaliscoMexicoUniversidad de Guadalajara, Centro Universitario de la Ciénega, Departamento de Ciencias Médicas y de la Vida, Ocotlan, Jalisco, Mexico; 5 Hospital Civil Fray Antonio AlcaldeGuadalajaraJaliscoMexicoHospital Civil Fray Antonio Alcalde, Servicio de Infectología Pediátrica, Guadalajara, Jalisco, Mexico; 6 Universidad de GuadalajaraUniversidad de GuadalajaraCentro Universitario de Ciencias de la SaludDepartamento de Biología Molecular y GenómicaGuadalajaraJaliscoMexicoUniversidad de Guadalajara, Centro Universitario de Ciencias de la Salud, Departamento de Biología Molecular y Genómica, Guadalajara, Jalisco, Mexico; 7 Universidad de GuadalajaraUniversidad de GuadalajaraCentro Universitario de Ciencias de la SaludDepartamento de Microbiología y PatologíaGuadalajaraJaliscoMexicoUniversidad de Guadalajara, Centro Universitario de Ciencias de la Salud, Departamento de Microbiología y Patología, Guadalajara, Jalisco, Mexico

**Keywords:** endocarditis, caspofungin, Candida albicans, liposomal amphotericin B

## Abstract

**BACKGROUND:**

Infective endocarditis is a disease characterised by heart valve lesions, which exhibit extracellular matrix proteins that act as a physical barrier to prevent the passage of antimicrobial agents. The genus *Candida* has acquired clinical importance given that it is increasingly being isolated from cases of nosocomial infections.

**OBJECTIVE:**

To evaluate the activity of caspofungin compared to that of liposomal amphotericin B against *Candida albicans* in experimental infective endocarditis.

**METHODS:**

Wistar rats underwent surgical intervention and infection with strains of *C. albicans* to develop infective endocarditis. Three groups were formed: the first group was treated with caspofungin, the second with liposomal amphotericin B, and the third received a placebo. In vitro sensitivity was first determined to further evaluate the effect of these treatments on a rat experimental model of endocarditis by semiquantitative culture of fibrinous vegetations and histological analysis.

**FINDINGS:**

Our semiquantitative culture of growing vegetation showed massive *C. albicans* colonisation in rats without treatment, whereas rats treated with caspofungin showed significantly reduced colonisation, which was similar to the results obtained with liposomal amphotericin B.

**CONCLUSIONS:**

The antifungal activity of caspofungin is similar to that of liposomal amphotericin B in an experimental model of infective endocarditis caused by *C. albicans*.

Infective endocarditis is a condition caused by pathogens such as bacteria and/or fungi that generates local inflammation, fibrin deposits, and platelets, known as fibrinous vegetations that are located in one or more valvular surface. Directly or inderectly, fibrinous vegetations can also affect other cardiac structures, such as chordae tendineae, mural endocardium, myocardium, and the pericardium (Durack & Beeson 1972).

In most cases of infective endocarditis, the permanence of microorganisms is favoured by the prior existence of an injury to the endothelium, a non-infected thrombus, prolapse or mitral regurgitation, aortic stenosis, rheumatic heart disease, systemic lupus erythematosus, or hypercoagulable states. However, some highly virulent bacteria have been reported to directly adhere to an intact endothelium (Slipczuk et al. 2014).

In recent years, *Candida albicans* has been one of the most frequently isolated pathogens in cases of nosocomial infections (Pfaller & Diekema 2010). This increase is due to the implementation of invasive procedures and the use of immunosuppressive drugs in transplant patients or immunocompromised patients (Silva et al. 2004, Klotz et al. 2007, Pfaller & Diekema 2010).

Although the presence of infective endocarditis caused by yeasts is rare, comprising 3% of all cases, up to 67% of these cases result in a fatal outcome in the absence of timely intervention. This high lethality rate is related to inherent properties of the fungal agent, such as the generation of biofilms, proteases, and adhesion factors that affect the immune status of the patient, as well as to the often-indiscriminate use of antifungals that can result in the failure of the treatment regimen (Ahmed et al. 2014, Thanavaro & Nixon 2014).

Antimicrobial treatment is essential to maintain high drug concentrations in serum and, thus, be able to target fibrinous vegetations. The use of liposomal amphotericin B and fluconazole have been suggested to ensure the death of the microorganisms and to improve the success of the treatment. However, the excessive and unnecessary use of antimicrobials for various therapeutic processes, combined with the increased selection of spread of resistance mechanisms in fungi, especially within the genus *Candida*, have necessitated the evaluation of new antifungals (Thanavaro & Nixon 2014).

Guidelines from the European Society of Cardiology and the American Heart Association for the management of *Candida* infective endocarditis include the administration of echinocandins, specifically caspofungin, preferably on the basis of blood cultures and sensitivity tests (Baddour et al. 2015, Habib et al. 2015). However, in addition to the other resistance mechanisms that these yeasts possess, biofilm production generates a barrier that affects the efficacy of many antimycotics. The aim of the study was to compare the effect of caspofungin to that of liposomal amphotericin B against *C. albicans* present in fibrinous vegetations in a rat model of infective endocarditis.

## MATERIALS AND METHODS

Three-month old, healthy female Wistar rats weighing between 250-300 g were used in our study. Infective endocarditis was induced with strains of *C. albicans*. A total of 18 female rats were divided into three groups, each consisting of six rats. Two groups received treatment: one group received caspofungin and the other liposomal amphotericin B. The third group, functioning as the control group, received a placebo treatment of 0.9% sterile saline solution.

In this study, we used the Durack and Benson model of infection modified by Marchetti to induce female Wistar rats with infective endocarditis, allowing for the development of vegetations. In brief, a polyethylene catheter (PE10, Clay Adams) was connected to a manometer and inserted into the left ventricle through the right carotid artery, under ketamine-xylazine anaesthesia. The catheter was indwelled throughout the experiment to induce thrombotic vegetation formation. This model has been validated for its reproducibility in evaluating the efficacy of new antimicrobials for this pathology (Durack & Beeson 1972, Marchetti et al. 2000).

*Isolation of a C. albicans strain* - The strain was obtained from a paediatric infected with *C. albicans* diseases in August 2008 and isolated in CHROMagar (BBL CHROMagar Candida Medium, Becton Dickinson GmbH.Heidelberg/Germany) *Candida* medium and characterised by end-point polymerase chain reaction (PCR) using a reference strain of *C. albicans* (ATCC 68548).

*DNA extraction* - The DNA extraction method was based on the protocol of Fujita et al. (2001). The isolated yeasts were cultured in Sabouraud dextrose agar for 24 to 48 h at 35ºC. Isolated colonies were incubated in 1 mL of Tris/EDTA buffer to a density of 0.5 McFarland standards. After centrifugation (5,000 *g* for 3 min) in a microcentrifuge tube, the pellet was resuspended in 0.1 mL of sorbitol (1 M) and EDTA (1M, pH 7.5) containing 5 µL of β-mercaptoethanol and 1 mg of lyticase (Sigma Chemical Co., St. Louis, MO). The mixture was incubated for 15 min at 37ºC. Finally, 5 µL of proteinase K solution (20 mg/mL) was added to the mixture, which was then incubated for 10 min in a water bath at 55ºC (Fujita et al. 2001).

*PCR* - To identify the strains used in the animal model, the following primers were used based on previous recommendations (Fujita et al. 2001): ITS1 (5’-TCCGTAGGTGAACCTGCG-3’), ITS3 (5’-GCATCGATGAAGAACGCAGC-3’), and ITS4 (5’-TCCTCCGCTTATTGATATGC-3’). These primers amplify conserved regions of 18S, 5.8S, and 28S, respectively rDNA. Amplification was performed in a final volume of 50 µL.

Amplification consisted of 30 cycles of an initial denaturation at 94ºC for 4 min, annealing at 55ºC for 30 s, extension at 72ºC for 1 min, and a final extension step at 72ºC for 4 min in a Perkin Elmer DNA Thermal Cycler 480.

*Electrophoresis* - The amplification products, along with a molecular weight marker (GeneRuler 50-bp DNA Ladder Fermentas®) were electrophoresed on agarose gels with 1.5% TBE buffer at a voltage of 300 mV for 45 min.

*In vitro analysis* - We evaluated the in vitro sensitivity of the previously mentioned isolated strain to caspofungin (Merck & Company, Rahway, NJ) and liposomal amphotericin B (NeXstar Pharmaceuticals Inc., Cambridge, UK). The strain was inoculated to induce the model of endocarditis. The inoculum was placed in a nutrient medium and was then incubated in RPMI (Roswell Park Memorial Institute) medium. The drug was titrated in order to determine the minimum inhibitory concentration according to the CLSI (Clinical and Laboratory Standards Institute) standards (Espinal & Pfaller 2007).

*Induction of experimental infective endocarditis* - Three-month old female Wistar rats weighing between 250 and 300 g were provided by the Bioterium of the University Centre for Health Sciences at the University of Guadalajara. Animals were exposed to 12-12-h light/dark cycles, with access to food and water ad libitum, according to the international guidelines for handling experimental animals. Animals were fasted for 24 h prior to surgery, and zoletil (tiletamine and zolazepam; Virbac) was administered at a dose of 10 mg per kg of body weight to induce and maintain anaesthesia during the surgical procedure. The intervention was performed after surgical asepsis, isolating the carotid artery and introducing a central catheter leading directly to the mitral valve and left ventricle (Marchetti et al. 2000). Then, 24 h later, we applied a fresh inoculum of *C. albicans* via the femoral vein.

*Inoculum preparation* - Previously identified colonies grown on Sabouraud dextrose agar were diluted in isotonic saline solution and measured at a wavelength of 620 nm with a spectrophotometer (Jenway, Sacramento, CA) to an optical density of 0.01, corresponding to 1.6 ´ 10^6^ colony-forming unit (CFU) per mL.

*Application of antimicrobial treatments* - Based on the minimum inhibitory concentration (MIC) of the isolated strain, we used the lowest recommended dosage in neonates to treat patients with infective endocarditis. Then, 24 h after inoculation, the treatment doses were administered intraperitoneally: caspofungin at 1 mg/kg of body weight, liposomal amphotericin B at 1 mg/kg of body weight, and the negative control group receiving 1 mL of isotonic saline. The three treatments lasted five days.

*Microbiological analysis* - The rats were sacrificed after 24 h following administration of the last dose on the 5th day, and the vegetations were dissected, weighed, and homogenised in 1 and 2 mL of saline solution, respectively, and serial dilutions were made in Sabouraud dextrose agar plates and incubated for 48 h at 36ºC. After incubation, the colonies were counted to determine the fungal density per gram of tissue or CFU by the following formula: density = Log (CFU/g vegetation) (Marchetti et al. 2000).

To validate that the isolated microorganism corresponded to *C. albicans*, the DNA from strain was subjected to the end-point PCR using the primers ITS1, ITS3, and ITS4 to identify conserved regions of 18S, 5.8S, and 28S rDNAs (Fujita et al. 2001). The ATTC 90028 reference strain was used for *C. albicans* as well as for another species of *Candida* (*C. glabrata*).

*Blood culture* - To estimate the fungal density (Jenway, USA) and to systemically observe the pharmacological effect, 3 mL of blood was collected via the femoral artery for cultivation in a nutrient broth (Versatreck Versa Diagnostics, Oakwood Village, OH, USA). Blood samples were incubated with a positive and negative control for 48 h to obtain readings at 620 nm on the spectrophotometer.

*Histology* - Tissue samples of vegetations were fixed in 4.5% formalin, embedded in paraffin, and then stained with a haematoxylin and eosin stain to examine the damage, to the vegetations inflammatory infiltrate, and presence of hyphae and/or yeast.

*Statistical analysis* - To determine the distribution of the data, a Shapiro-Wilk test was performed where all of the data showed an abnormal distribution. Analysis of the antifungal fibrinous density of vegetations was performed by nonparametric analysis using the Mann-Whitney test; values were considered significant at p < 0.05. The qualitative variables are presented as proportions and statistical analysis by chi-square using descriptive statistics. The statistical software used was SPSS 21.

## RESULTS

**Fig. 1:**
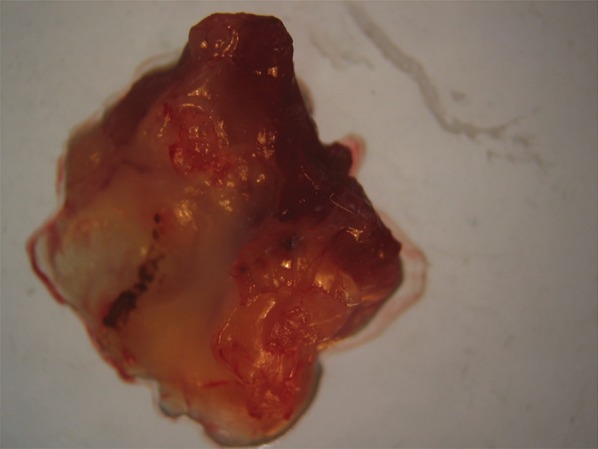
: isolated heart valve from rats treated with antimicrobial agents and infected with *Candida albicans*, showing the presence of vegetations (arrow).

Analysis of the in vitro *C. albicans* inoculum sensitivity showed an MIC of 0.625 µg/mL for caspofungin and 1 µg/mL for liposomal amphotericin B. All animals developed vegetations characteristic of infective endocarditis (Fig. 1).

**Fig. 2:**
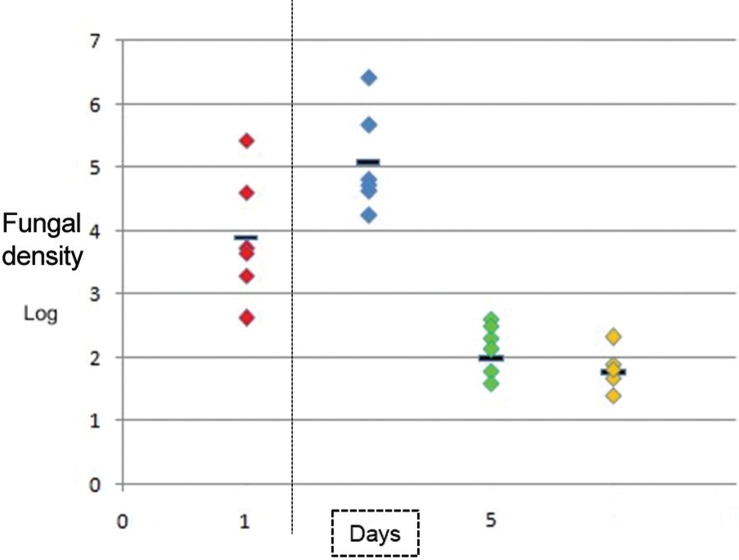
: comparative representation of fungal densities (in logarithmic scale) of fibrinous vegetations, where the fungal densities of rats without treatment are shown in group 1 (red). Group 2 represents mycotic densities of rats without treatment and a 5-day evolution of the disease (blue); group 3 represents the mycotic densities of vegetations from rats treated with caspofungin (green); and group 4 represents the mycotic densities of vegetations from rats treated with liposomal amphotericin B (yellow).

Fig. 2 shows an increase in fungal density (in a logarithmic scale of vegetations) in rats infected 24 h and five days after inoculation, with a vegetations average of 3.89 CFU/g tissue at 24 h and an average of 5.08 CFU/g tissue (p < 0.05) at five days after inoculation. Fig. 2B shows the differences in fungal density log that were present in the culture homogenates of fibrinous vegetations in the different study groups.

The values for the blood culture samples were obtained for the blood culture samples were statistically different (p < 0.05) between the group treated with caspofungin (or with the group treated with liposomal amphotericin B) and the untreated rats. Meanwhile, no statistically significant differences were found between the caspofungin-treated group and the liposomal amphotericin B-treated group (Table).

**TABLE:** Statistical analysis of the average absorbance of blood cultures from untreated rats, rats treated with caspofungin, and rats treated with liposomal amphotericin B compared to a positive control *Candida albicans* (ATCC 68548) inoculated without treatment

Group	Untreated	Caspofungin	Liposomal amphotericin B
Average absorbance	0.878	0.230	0.251
Positive control	0.865		

**Fig. 3:**
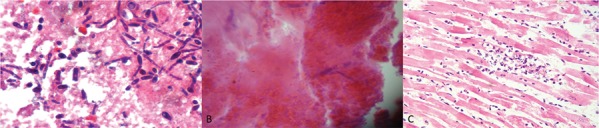
: histological samples of the study groups that show the following: (A) aseptic vegetation; (B) vegetation in rats treated with caspofungin; and (C) vegetation in rats treated with liposomal amphotericin B.

In Fig. 3, histological changes in vegetations between the different groups are shown: (A) septic vegetation in untreated animals, which shows an inflammatory infiltrate with fibrin deposition and an abundance of yeast and pseudohyphae; (B) vegetation in caspofungin-treated rats, which shows evidence of inflammation with a decreased number of hyphae but present in a deformed state; (C) vegetation in rats treated with liposomal amphotericin B, where evidence of cell damage and low yeast abundance are shown.

**Fig. 4:**
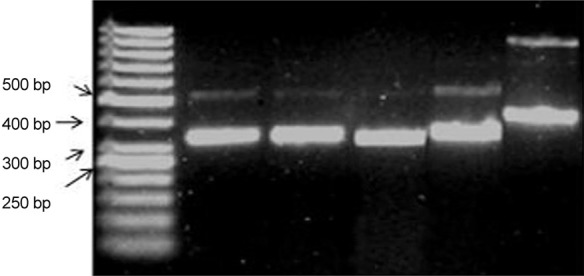
: image from agarose gel electrophoresis of DNA fragments amplified using end-point polymerase chain reaction, which shows the molecular marker of 50 bp in lane 1. Lane 2 and 4 show isolated strains of vegetations, lane 5 shows an ATTC 90028 strain, and lane 6 shows nonspecific amplicons of a non-*albicans Candida* species.

PCR-based identification of the pathogenic agent is shown in Fig. 4; all isolated microorganisms were identified as *C. albicans*.

## DISCUSSION

Infective endocarditis is generally caused by microorganisms that are capable of generating biofilm and primarily occurs in patients undergoing invasive procedures, such as catheterisation and in subjects with valvular pathologies (Kojic & Darouiche 2004). *C. albicans* is frequently reported as a cause of nosocomial infection resulting from improper handling of catheters and other factors, including the prior use of antibiotics, steroid use, parenteral nutrition, and abdominal procedures. The invasive capacity of *C. albicans* is due to its cell wall composition, as well as by the enzymes that it expresses, including proteases and inflammatory cytokines (Filler & Sheppard 2006). Moreover, *C. albicans* is capable of biofilm formation, which serves as a barrier to block the penetration of antimicrobial agents (Hernández-Cañaveral et al. 2009) and confers resistance to several antifungals, resulting in a high rate of mortality for patients with endocarditis.

The infective endocarditis model showed a high degree of reliability and viability in assessing the efficacy of antimicrobial agents in infective endocarditis. However, while the activity of antifungal agents in cultures is widely used (Habib 2006), there is no guarantee of its in vivo effect in endocarditis caused by bacteria capable of biofilm formation (Hernández-Cañaveral et al. 2009).

Histological characterisation of the vegetations present in animals were consistent with those reported by Durack and Beeson (1972), who initially standardised a technique for induction of infective endocarditis. Similarly, the results of our histological evaluation are consistent with those reported by Marchetti et al. (2000), who introduced a method for calculation of mycotic density in order to compare the densities of microorganisms present in vegetations. Our results show the expected therapeutic efficacy of both caspofungin and liposomal amphotericin B for reducing fungal density. These results are similar to those obtained by Habib (2006). On the other hand, doses used in this study differ from the published MIC obtained in vitro, because our studies showed an increased sensitivity to caspofungin compared to liposomal amphotericin B. However, no differences were found in the microorganism density of vegetations treated with caspofungin compared to those treated with liposomal amphotericin B. Blood cultures also showed decreased fungal density of these microorganisms between the untreated group and the groups receiving antifungal treatment. In agreement with previous reports, our results demonstrate the efficacy and systemic activity of both agents administered intraperitoneally (Gulati et al. 1998). However, it should be noted that although the culture can be negative once the patient concludes treatment, the microorganism itself is not eliminated in vegetations (Pappas et al. 2009). Decision making with respect to surgical intervention is complex and involves several factors; among which, the following factors are particularly important: vegetation size, abscess formation, failure to respond to treatment, heart failure or embolism, age, and comorbidities. In endocarditis caused by a fungal infection, timely surgical intervention dramatically improves the survival outcome (Baddour et al. 2015).

We found that the rat model replicated fungal-infected vegetation. Moreover, it proved to be useful to examine the efficacy of these pharmacological agents in the short term, because the mortality of the animals was increased considerably after five days following inoculation. However, reducing the amount of inoculum would have been insufficient to show infected vegetation, and/or increasing the time of treatment would not have allowed for a comparison of antifungal differences in vegetations given the higher animal mortality rate. This could be interpreted as a limitation when extrapolating the disease outcome to humans; however, our study provides an important first step in demonstrating the effectiveness of caspofungin treatment in a rat mode.

Although fungal density did not reach negative levels (zero), significant differences between the treated and untreated groups were found. This demonstrates that both treatments have an antimicrobial effect against *C. albicans* in an experimental model of infective endocarditis. On the other hand, several studies have shown that amphotericin (non-liposomal) alone does not have an antifungal effect in animal models of fungal endocarditis (Pappas et al. 2009). However, the liposomal formulation of amphotericin managed to overcome the barriers generated by the microorganism. Moreover, this formulation has the additional advantage of having less serious side effects in patients (Gulati et al. 1998).

Both antifungal agents caused changes in the colony morphology of the fungus compared to the vegetations observed in the untreated group. Histological studies of fibrinous vegetations provided useful data. In rats treated with caspofungin, not only was the colony size reduced, but the remaining hyphae showed deformation, showing damage to the membrane. In contrast, in rats treated with liposomal amphotericin B, the colony size was reduced, but no deformation was observed, suggesting that the antifungal effect of caspofungin differs from that of liposomal amphotericin B. Indeed, caspofungin was shown to affect synthesis of Becton Dickinson-glucan synthase by altering the fungal cell wall, whereas amphotericin affects membrane permeability (Gulati et al. 1998). To our knowledge, these morphological features have not been previously described in the literature.

In order to ensure the validity of the study and that no contaminants were present, yeasts were isolated and typified by PCR, confirming the clonal characterisation of *C. albicans* in rats.

This study confirms the antifungal activity of caspofungin and liposomal amphotericin B in an experimental animal model of infectious endocarditis caused by *C. albicans*. Consequently, these findings provide precedence to the effectiveness of these agents in endocarditis. Because of the high cost of clinical trials, this experimental model represents an excellent opportunity to evaluate treatment in infective endocarditis.

## References

[B1] Ahmed A, Azim A, Baronia AK, Marak KRSK, Gurjar M. Risk prediction for invasive candidiasis. Indian J Crit Care Med. 2014; 18(10): 682-8. 10.4103/0972-5229.142178 PMC4195199 25316979

[B2] Baddour LM, Wilson WR, Bayer AS, Vance G, Fowler Jr VG, Tleyjeh IM, et al. Infective Endocarditis in adults: diagnosis, antimicrobial therapy, and management of complications. A scientific statement for healthcare professionals from the American Heart Association. Circulation. 2015; 132(15): 1435-86. 10.1161/CIR.0000000000000296 26373316

[B3] Durack DT, Beeson PB. Experimental bacterial endocarditis. I: colonization of a sterile vegetation. Br J Exp Pathol. 1972; 53(1): 44-9. PMC2072378 5014243

[B4] Espinal AV, Pfaller M. Susceptibility test methods: yeast and and filamentous fungi. In: Murray PR, Baron EJ. Manual of clinical microbiology. 9th ed. Vol. II. Washington (DC): ASM Press; 2007. p. 1975-7.

[B5] Filler SG, Sheppard DC. Fungal invasion of normally non-phagocytic host cell. PLoS Pathog. 2006; 2(12): 1099-105. 10.1371/journal.ppat.0020129 PMC1757199 17196036

[B6] Fujita SI, Senda Y, Nakaguchi S, Hashimoto T. Multiplex PCR using internal transcribed spacer 1 and 2 regions for rapid detection and identification of yeast strains. J Clin Microbiol. 2001; 39(10): 3617-22. 10.1128/JCM.39.10.3617-3622.2001 PMC88398 11574582

[B7] Gulati M, Bajad S, Singh S, Ferdous AJ, Singh M. Development of liposomal amphotericin B formulation. J Microencapsul. 1998; 15(2): 137-51. 10.3109/02652049809006844 9532520

[B8] Habib G, Lancellotti P, Antunes MJ, Bongiorni MG, Casalta JP, del Zotti F, et al. The 2015 ESC Guidelines for the management of infective endocarditis. Eur Heart J. 2015; 36(44): 3036-7. 10.1093/eurheartj/ehv488 26590409

[B9] Habib G. Management of infective endocarditis. Heart. 2006; 92(1): 124-30. 10.1136/hrt.2005.063719 PMC1861013 16365367

[B10] Hernández-Cañaveral I, Becerra G, Jiménez-Cordero A, Michel J-B, Plascencia A, Domínguez-Hernández M. *Candida albicans* isolated from human fungaemia induces apoptosis in an experimental endocarditis model. Mem Inst Oswaldo Cruz. 2009; 104(6): 858-61. 10.1590/s0074-02762009000600006 19876556

[B11] Klotz SA, Chasin BS, Powell B, Gaur NK, Lipke PN. Polymicrobial bloodstream infections involving *Candida* species: analysis of patients and review of the literature. Diagn Microbiol Infect Dis. 2007; 59(4): 401-6. 10.1016/j.diagmicrobio.2007.07.001 17888612

[B12] Kojic EM, Darouiche RO. *Candida* infections of medical devices. Clin Microbiol Rev. 2004; 17(2): 255-67. 10.1128/CMR.17.2.255-267.2004 PMC387407 15084500

[B13] Marchetti O, Entenza JM, Sanglard D, Bille J, Glauser MP, Moreillon P. Fluconazole plus cyclosporine: a fungicidal combination effective against experimental endocarditis due to *Candida albicans*. Antimicrob Agents Chemother. 2000; 44(11): 2932-8. 10.1128/aac.44.11.2932-2938.2000 PMC101583 11036003

[B14] Pappas PG, Kauffman CA, Andes D, Benjamin Jr DK, Calandra TF, Edwards Jr JE, et al. Clinical practice guidelines for the management of candidiasis: 2009 Update by the Infectious Diseases Society of America. Clin Infect Dis. 2009; 48(5): 503-35. 10.1086/596757 PMC7294538 19191635

[B15] Pfaller MA, Diekema DJ. Epidemiology of invasive mycoses in North America. Crit Rev Microbiol. 2010; 36(1): 1-53. 10.3109/10408410903241444 20088682

[B16] Silva V, Díaz MC, Febré N, Chilean Invasive Fungal Infections Group. Invasive fungal infections in Chile: a multicenter study of fungal prevalence and susceptibility during a 1-year period. Med Mycol. 2004; 42(4): 333-9. 10.1080/13693780410001657153 15473358

[B17] Slipczuk LJ, Codolosa N, Davila C, Romero-Corral A, Yun J, Pressman GS, et al. Infective endocarditis epidemiology over five decades: a systematic review. PLoS ONE. 2014; 9(10): e111564. 10.1371/journal.pone.0082665 PMC3857279 24349331

[B18] Thanavaro KL, Nixon JV. Endocarditis 2014: an update. Heart Lung. 2014; 43(4): 334-7. 10.1016/j.hrtlng.2014.03.009 24780242

